# Optimising acute stroke pathways through flexible use of bed capacity: a computer modelling study

**DOI:** 10.1186/s12913-022-08433-0

**Published:** 2022-08-20

**Authors:** Richard M. Wood, Simon J. Moss, Ben J. Murch, Christos Vasilakis, Philip L. Clatworthy

**Affiliations:** 1 Bristol, North Somerset and South Gloucestershire ICB, National Health Service, South Plaza, Marlborough St, BS1 3NX Bristol, United Kingdom; 2 School of Management, University of Bath, Claverton Down, BA2 7AY Bath, United Kingdom; 3 North Bristol Trust, National Health Service, Southmead Road, BS10 5NB Bristol, United Kingdom

**Keywords:** Stroke Care, Stroke Centralisation, Demand and Capacity, Simulation

## Abstract

**Background:**

Optimising capacity along clinical pathways is essential to avoid severe hospital pressure and help ensure best patient outcomes and financial sustainability. Yet, typical approaches, using only average arrival rate and average lengths of stay, are known to underestimate the number of beds required. This study investigates the extent to which averages-based estimates can be complemented by a robust assessment of additional ‘flex capacity’ requirements, to be used at times of peak demand.

**Methods:**

The setting was a major one million resident healthcare system in England, moving towards a centralised stroke pathway. A computer simulation was developed for modelling patient flow along the proposed stroke pathway, accounting for variability in patient arrivals, lengths of stay, and the time taken for transfer processes. The primary outcome measure was flex capacity utilisation over the simulation period.

**Results:**

For the hyper-acute, acute, and rehabilitation units respectively, flex capacities of 45%, 45%, and 36% above the averages-based calculation would be required to ensure that only 1% of stroke presentations find the hyper-acute unit full and have to wait. For each unit some amount of flex capacity would be required approximately 30%, 20%, and 18% of the time respectively.

**Conclusions:**

This study demonstrates the importance of appropriately capturing variability within capacity plans, and provides a practical and economical approach which can complement commonly-used averages-based methods. Results of this study have directly informed the healthcare system’s new configuration of stroke services.

**Supplementary Information:**

The online version contains supplementary material available at 10.1186/s12913-022-08433-0.

## Background

Effectively managing demand and capacity is essential for hospitals and healthcare systems. The challenge is to provide the appropriate amount of capacity for services whose demand can be volatile and difficult to predict. This is especially important for non-elective activity, where the number of unplanned presentations can be influenced by the largely inestimable effect of factors such as the weather, road conditions, and major public events [1]. For admitted care, the problem is compounded by variability in the patient arrival rate and length of stay [2]. Further complexities exist for pathways requiring continuous care, where unbalanced demand and capacity can cause delayed admissions which propagates pressure to upstream services [3]. 

The consequences of this can be severe. Approximately 1.35 million acute bed days were lost in England in financial year 2019 due to the unavailability of downstream care [4], at an estimated cost of £900 m [5]. However, this does not account for the consequent effects on the wider hospital apparatus. With delays to discharge increasing length of stay, the acute bed base is put under greater pressure; elevating the likelihood of elective procedure cancellations and restricting the ability to admit patients from the Emergency Department, which in turn causes overcrowding and ambulance offload delays [6, 7]. Furthermore, delays to discharge have been associated with reduced patient functional and cognitive independence, which additionally may increase the remedial or longer-term burden placed on downstream services [8].

 For centralised hyper-acute stroke services, poor capacity management carries the risk of an additional and more fundamental problem. The core benefit of centralisation is the ability for patients to readily access specialist resources and treatments (such as thrombectomy) which can reduce the damage caused by stroke and improve long term prognoses [9]. However, if there is no available capacity in the hyper-acute stroke units (HASUs) designed to deliver such care, then the relatively short window to intervene may expire. Following the recognised merits of establishing HASUs in London and Manchester [10], there is an expectation for all health and care systems in England to transition to centralised hyper-acute models [11]. This includes the Bristol, North Somerset and South Gloucestershire (BNSSG) system which is the setting of this study and covers a one million population in South West England. At the time of writing, the BNSSG system has, after many years of planning, approved a final Decision-Making Business Case on proposals for its future centralised stroke service, including a detailed assessment of the number of beds required along the patient pathway. This paper describes the approach taken in determining such assessments, through novel consideration and quantification of the *flex capacity* requirements for the hyper-acute, acute and rehabilitation units.

### Flex capacity

It is typical for healthcare organisations, both in the UK and further afield, to estimate capacity requirement through average patient arrival rate and length of stay [2, 12]. Yet such approaches are inherently flawed since they do not appreciate the afore-mentioned sources of variability which, unchecked, can lead to discharge delays, large waits, and patients being turned away. Previous studies of stroke pathways have revealed that averages-based, or ‘deterministic’, methods can under-estimate capacity requirement by up to 40% [13, 14]. While ‘stochastic’ methods – often computer-based models capturing variation in arrivals, lengths of stay, and discharge/transfer delays – offer improved accuracy through a better conceptual fit to the problem, there is not always the necessary technical competency to deploy such approaches within health services, nor a level of familiarity to comprehend their outputs [15].

 Given widespread use of averages-based approaches, the objective of this study was to investigate the methodological and practical extent to which their outputs could be complemented in the interests of achieving a more robust assessment of capacity needs. To this end, and with application to the centralisation of hyper-acute stroke services in the BNSSG healthcare system, consideration was given to the *flex capacity* required in addition to the averages-based number of beds in order to mitigate any negative consequences of peaks in demand. Flex capacity is defined as additional beds which are available at short notice but are not part of the routinely allocated bed base for that ward. They may be beds which are used for non-stroke patients, but which can be rapidly cleared if a stroke patient arrives, or beds for which there is no clinical resource routinely allocated, but such resource can be mobilised at short notice (e.g. bringing in extra nurses and physicians on an on-call basis, or from other duties). 

**Fig. 1:**
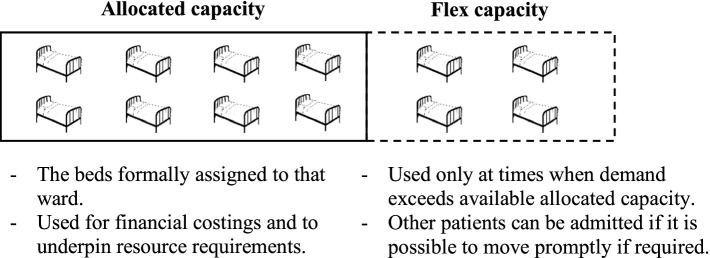
Outline of differences between *allocated capacity* and *flex capacity*

Following established practice, the *allocated capacity* of units on the pathway was calculated through a deterministic averages-based approach. Familiarity with this approach allowed the healthcare system to make confident decisions about finance and workforce requirements. More advanced stochastic methods were then used to determine the additional *flex capacity* requirement, in terms of the frequency and magnitude of outreach into adjacent pools of clinically appropriate stroke unit beds (Fig. 1). Such flexible use of hospital capacity has economic benefits with the alternative being the dedication of large amounts of allocated capacity to each unit, much of which would be unused much of the time [16]. Despite the benefits of flexible capacity use, the authors could find no published study to date in which the quantification of flex capacity has been objectively approached.

### Modelling the stroke pathway

Modelling contributes to health service planning by allowing the assessment of alternative scenarios, strategies and resource configurations that cannot be readily, feasibly or safely examined in real life [17]. Given the above-mentioned complexities of managing capacity along the stroke pathway, a number of modelling studies have been reported in the literature. Among the first published studies, Heinrichs et al. [18] used computer simulation to model patient flow for acute stroke services at a Dutch hospital, concluding that “*simulation models provide a powerful tool for supporting decision making with regard to resource planning*”. Quaglini et al. [19] found similar potential, in modelling activities at an Italian stroke unit “*to detect bottlenecks in the care delivery organisation and to find the optimal resource allocation*”. Bayer et al. [20] used simulation to examine the impact of improved telehealth and acute care on bed day requirement. McClean et al. [21] focused on the effect of discharge delays on bed occupancy and required capacity. Finally, Monks et al. [13] performed various ‘what if’ analyses and estimated that the number of stroke presentations facing delayed admission would reduce from 1 in 7 to 1 in 50 if acute capacity is increased from 10 to 14 beds.

A smaller number of modelling studies have considered the centralisation of stroke services. Lahr et al. [22] used simulation to compare performance of decentralised and centralised pathways, finding the latter “*substantially lowers mean annual costs per patient*”. In their subsequent work, having moved to a centralised configuration, they used simulation to further optimise the acute care pathway [23]. Hunter et al. [24] used a model of patient flow as part of their economic evaluation of the centralisation of stroke services in London and Manchester in 2010, with mixed results leading to their tenuous conclusion that centralisation “*may result in a net health benefit to a region*”. Earlier modelling to support stroke centralisation in the BNSSG healthcare system has also demonstrated the value of computer simulation, with the capacity requirements of the current decentralised pathway compared to that of a centralised ‘future state’ option being considered at the time [14].

However, in none of these studies is *flex capacity* formally considered, despite some acknowledgement of the problems its use may address. For instance, Heinrichs et al. [18] recognised “*the trade-off between regular under-staffing and a low bed occupancy rate*” (indeed, Wood & Murch [14] calculated that to ensure no more than 1 in 100 stroke presentations face delayed admission required a mean HASU occupancy of just 52%). This is the very issue for which flexible capacity management can offer a potential solution, allowing alternate use of capacity that would otherwise be under-utilised provided operational plans are in place to make this flex capacity available when required. In terms of related research, there are some parallels with the modelling of ‘surge capacity’ in the field of disaster management [25]. In seeking to bridge the gap to routine patient flow, Asplin et al. [26] capture demand surges in modelling an emergency department. However, they do not derive the frequency and magnitude of additional capacity requirements over and above that which is allocated as standard. These examples underline the paucity of applied research in this area, thus highlighting the need and opportunity for new studies.

## Methods

### Study setting

**Fig. 2:**
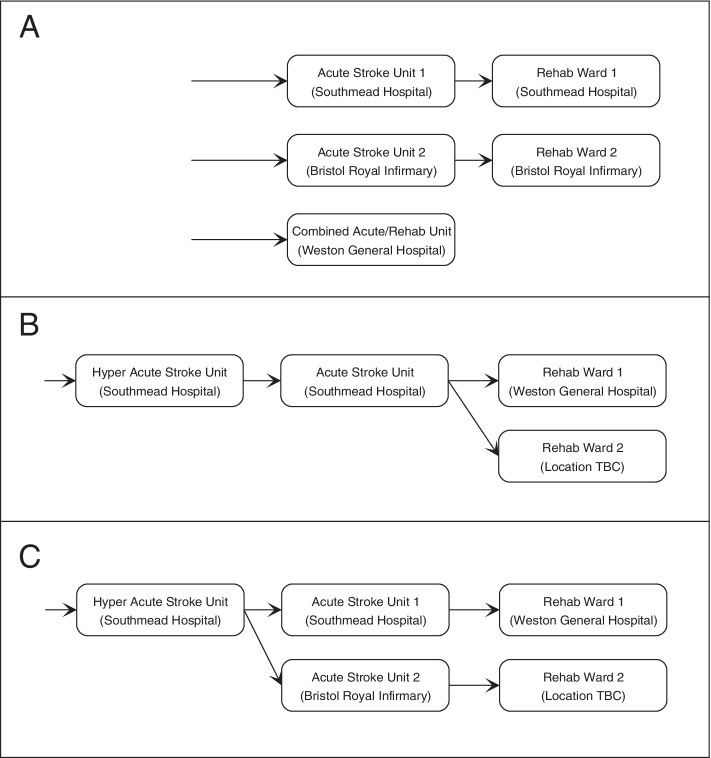
Specification of (**A**) current decentralised pathway, and proposed centralised stroke pathway options involving one hyper acute stroke unit, either one (**B**) or two (**C**) acute stroke units, and two rehabilitation wards

The Bristol, North Somerset and South Gloucestershire (BNSSG) care system is a major health economy located in South West England. It has a single Integrated Care Board (ICB) that oversees the organisation and procurement of taxpayer-funded healthcare activity for the approximate one million resident population. Acute stroke care is decentralised and is commissioned from the three hospitals operating within the BNSSG geography (Fig. 2A). In the terminology given by NHS England, in their National Stroke Service Model [27], this accounts for one Comprehensive Stroke Centre (CSC) and two Acute Stroke Centres (ASCs). Ongoing rehabilitation is commissioned from the single community services provider, and two of the three hospitals provide home-based Early Supported Discharge (ESD) services.

 The BNSSG population has been growing at approximately 0.7% per year, with a higher growth rate of 1% per year in the number of people aged 55 or over. In 2019/20, there were approximately 1,400 hospital admissions for stroke according to Sentinel Stroke National Audit Programme (SSNAP) data [28], which had been increasing by around 2% per year over the preceding five-year period. In 2019/20 there were an estimated 19,000 people living in the BNSSG region who had previously been diagnosed with stroke. In the same year, the CSC performed 124 mechanical thrombectomy procedures. Further descriptive information on the BNSSG population and those affected by stroke is available at [29] (Sect. 4). 

For a number of years, the BNSSG health and care system has sought to centralise hyper-acute stroke care and has been working towards a Full Business Case setting out the new service specification and the attendant benefits. A key part of this effort has been the estimation of required capacity along the envisaged pathway. This has been approached through computer modelling, the outputs of which have featured in an earlier Outline Business Case for a considered pathway involving a single hyper-acute stroke unit (HASU) at the CSC, a single acute stroke unit (ASU) for ongoing acute care, also at the CSC, and two stroke rehabilitation wards for patients who are sufficiently stable to be discharged from acute hospital care [14]. The proposals included development of what would later be known as an Integrated Community Stroke Service, providing ESD services and ongoing stroke rehabilitation and care equitably across the region and maximising opportunities for home-based care [30].

Since then, the Programme Board charged with overseeing centralisation has decided that an option of two ASUs should also be considered alongside the ‘preferred’ option involving one ASU (Figs. 2B and 2C). The second ASU, if approved, would provide care from the point of discharge from the HASU at the CSC to the point at which patients no longer require acute hospital care and can be either discharged home or transferred to one of the rehabilitation wards. Additionally, the Board decided, given concerns on operationally and financially infeasible modelled average occupancy rates (just 52% for the HASU), that bed requirement should be considered in terms of those beds specifically *allocated* to the stroke pathway and those available within the wider stroke bed base that could be used *flexibly* at times of peak demand. Allocated capacity would be calculated using averages-based approaches familiar to service planners and Board members, with flex capacity derived from use of the appropriate stochastic modelling methods. Results would be required as part of the proposal submitted for public consultation as well as the final Decision-Making Business Case.

### Systems modelling and computer simulation

Computer simulation was used to model patient flow along the stroke pathway. Simulation was preferred to an analytical (mathematical) solution given its flexibility to different parameters and pathway configurations. Modelling was performed through a conceptually-appropriate (open-source) computer simulation tool [31], purpose-built for use in the healthcare setting, and freely available to others on GitHub [32]. The tool implements the established ‘three phase’ method to stochastic simulation [33], in which separate ‘discrete’ events are generated according to a schedule in which the next unconditional event is executed alongside any associated conditional events. The first type of unconditional event is a patient arrival, i.e. a stroke presentation at the CSC for admission to the HASU. If this occurs at a time when there is sufficient HASU capacity, then the generated conditional event is HASU admission. Otherwise, the patient must wait. The second type of unconditional event is a patient becoming ready for discharge from one of the pathway units. If there is available capacity at the discharge destination then they are discharged, and any upstream waiting patient is admitted in their stead. Otherwise they remain at their current location until downstream capacity becomes available. The schedule is updated at each iteration and events continue until the end of the simulation period is reached. 

Results were obtained by performing multiple replications of the simulation, each with a different random number seed used to generate the timing of patient arrivals and the lengths of stay at each unit. The former was sampled from a Poisson distribution (assuming independence of one arrival to another) and the latter from the most appropriate distribution for that unit (see next sub-section). For each simulation, 1500 replications were performed, each for one year in duration and with a warm-up period of 100 days (this is used to ensure the system reaches steady-state, before the period for which results are captured). For more information regarding the simulation, see Supplementary Material A.

### Data and calibration

The daily rate of arrival of patients with suspected stroke on the HASU at the CSC was determined from a combination of sources, including local ambulance trust data, patient-level SSNAP data [28] and local business intelligence data for the mechanical thrombectomy service. This was increased by projected growth in suspected stroke ambulance conveyances from 2019/20 of 5%. Beyond the total patient arrival rate, there was limited empirical data available to support model calibration, especially regarding the HASU, given that many acute stroke services were non-centralised at the time of the study. Where possible, the relevant information was used [28, 34], which included service-level SSNAP data from centres with centralised hyperacute stroke services (London HASUs). Downstream parameters were estimated using local data obtained from hospital Patient Administration Systems (PAS) and from a 2017 service evaluation. This service evaluation was performed specifically to support the capacity modelling. Detailed information was captured that is not collected within the PAS, e.g. date medically fit for discharge – useful in calculating length of stay until the point of discharge readiness. All model parameters for both Options 1 and 2 are contained in full within Supplementary Material B, including the specific source of information used for estimation.

## Results

**Fig. 3:**
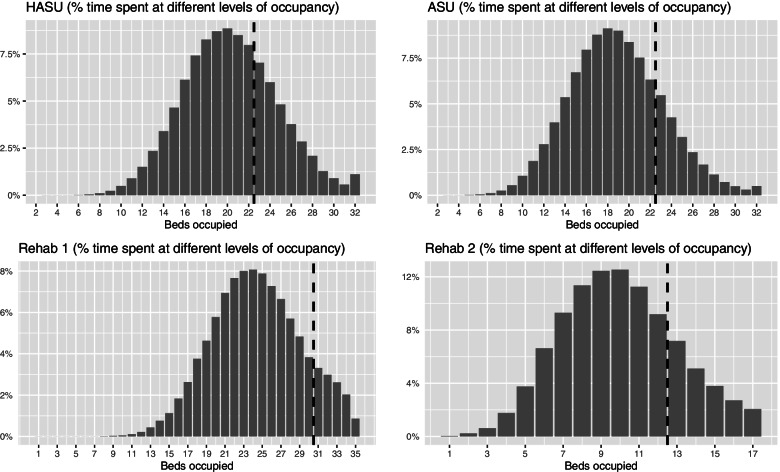
Modelled bed occupancy for the proposed future centralised stroke service, under the (preferred) Option 1. The dashed vertical lines represent the demarcation between allocated and flex capacity utilisation

The modelled performance measures are summarised in Table 1 for the two pathway options under consideration, with ward occupancy distributions provided in Fig. 3 (Option 1 only). For both options, it is estimated that allocated HASU capacity will be sufficient approximately 70% of the time. Average flex capacity requirement is approximately one bed, with the full ward capacity of 32 beds (i.e. including the maximum 10 flex beds) being used 1% of the time. Splitting the ASU capability to two units is not shown to adversely affect performance, provided the additional two allocated beds. Indeed, with the additional 18 flex beds, the likelihood of reaching full flex capacity is reduced from 0.5% to zero. Under both options, allocated capacity is used approximately 88% and 80% of the time for Rehab units 1 and 2 respectively. For Rehab unit 2, total capacity is reached approximately 2% of the time, indicating the potential need for a higher limit. However, the consequential ASU discharge delays that would occur at these times does not suggest a significant problem given the negligible amounts of time the ASU(s) themselves are at full capacity (a marker, in turn, for HASU fluidity). 

With no flex capacity available in the Rehab units (an early assumption of the stroke centralisation Programme Board), there are greater delays to discharge to these units and so greater pressure on upstream services (Table 1 and Supplementary Material C). Under the preferred Option 1, the amount of time the HASU is within allocated capacity reduces from approximately 70% to 64%, with an 80% to 56% reduction for the ASU. This leads to a greater average flex capacity requirement (1.7 and 2.6 beds c.f. 1.1 and 0.7 beds) and a greater amount of time at full flex capacity (6.5% and 12.8% c.f. 1.1% and 0.5%). Under Option 2, the large amount of ASU flex capacity absorbs most of the pressure, thus insulating the HASU from much of the effect. Should there exist no flex capacity along the entire modelled pathway, then pressures are greater still (Supplementary Material D). This highlights the inadequacy of averages-based approaches, if used alone for the calculation of capacity requirement.

**Table 1:** Modelled performance results for the proposed future centralised stroke service, involving either one (Option 1; preferred option) or two Acute Stroke Units (Option 2). Results also included for both options with no flex for the Rehab units

Option	Unit	Allocated capacity, beds (total with flex)	Mean total occupancy, beds	Time within allocated capacity, %	Mean flex capacity required, beds	Time at full allocated and flex capacity, %
1	HASU	22 (32)	20.3	69.6	1.1	1.1
	ASU	22 (32)	18.9	79.9	0.7	0.5
	Rehab 1	30 (35)	24.4	88.2	0.3	0.9
	Rehab 2	12 (17)	9.9	79.1	0.5	2.1
2	HASU	22 (32)	20.3	69.5	1.0	1.1
	ASU 1	15 (32)	11.2	89.2	0.3	0.0
	ASU 2	9 (20)	7.6	75.9	0.6	0.0
	Rehab 1	30 (35)	24.5	87.9	0.3	0.9
	Rehab 2	12 (17)	9.9	79.8	0.5	1.8
***With no flex for Rehab units***						
1	HASU	22 (32)	21.2	63.6	1.7	6.5
	ASU	22 (32)	22.2	55.6	2.6	12.8
	Rehab 1	30 (30)	24.3	100.0	-	93.7
	Rehab 2	12 (12)	9.7	100.0	-	73.9
2	HASU	22 (32)	20.4	68.7	1.2	1.6
	ASU 1	15 (32)	13.5	71.6	1.3	0.4
	ASU 2	9 (20)	9.2	58.6	1.3	1.9
	Rehab 1	30 (30)	24.4	100.0	-	93.5
	Rehab 2	12 (12)	9.7	100.0	-	74.2

## Discussion

### Strengths and limitations

The distinction between different types of bed capacity has not been investigated in the stroke modelling literature to date. This creates a gap between theory and practice, which is partly addressed through this study. In doing so, we show how appropriate consideration to both *allocated* and *flex* capacity can produce a bed plan that is sufficiently appreciative of patient safety and financial sustainability to be acceptable to both clinicians and hospital management. To maximise acceptability of the model by all stakeholders, we established its face validity through regular presentation and communication of its structures, inputs, and outputs. This took place over many years at monthly meetings of the Stroke Programme Board and fortnightly meetings of a sub-group concentrating on patient activity and finance matters. In addition, the regional Clinical Senate undertook an independent review prior to public consultation, finding that “*the business case is informed by robust capacity and demand modelling*” and “*the inclusion of flex capacity within the modelled was considered to be valuable*”. The panel also “*explored the clinical assumptions on which the model is based and can confirm that these are realistic*”, thus providing further support to model validation [35]. 

As with any modelling studies, a number of simplifying assumptions were necessary. One key assumption is that flex capacity is always available when required – this is unlikely fully true, especially during times of severe system pressure (e.g. winter spikes) when multiple specialties seek to outreach into other bed pools. In this respect, the results presented here represent the best-case scenario in terms of the benefits that can be gained from using flex capacity in the considered stroke pathways. However, the inclusion of a defined number of beds identified as flex capacity, along with an estimate of how often these beds will be required, permits development of operational plans to make these beds available when needed. Hospitals have contingency arrangements to make ‘escalation capacity’ available at times of severe pressure, but activation of these arrangements impacts other services such as elective work and has financial implications such as the need for agency workforce. The quantitative outputs from the modelling mean that these plans can be made in a way to minimise disruption of services and manage the associated financial consequences.

Another key assumption is that treatment in a flex bed has no effect on treatment quality. Where beds are staffed by a highly specialist workforce, such as on hyper-acute stroke units, availability of beds does not necessarily translate to maintained quality of care. However, flex capacity can be used to help ensure that patients remain within a specialist unit and plans can be made to staff the additional capacity in a safe manner, minimising the impact on quality of patient care. In this study, the maximum size of a ward (32 beds) has been used to define the upper limit of flex capacity, so that even when this capacity is in full use, patients can remain on the stroke unit and arrangements can be made to manage the specialist workforce appropriately. 

Other potentially limiting assumptions include the use of a homogenous Poisson process for stroke arrivals, where realistically there is some amount of cyclicality in the arrival rate based on the hour in day. The effect of this could be both an over- and under-estimation of bed occupancy, depending on the time of day. Assumptions relating to the model parameters should also be acknowledged. While the Programme Board had overseen and approved model calibration on the basis of the best information available at the time (see *Methods; Data and calibration*), there remained particular uncertainty for some of the parameters, given the lack of precedence for such a reorganisation within the system. Specifically, uncertainty related to the extent to which the estimated lengths of stay and fixed transfer delays (Supplementary Material B) would be achieved. There was also an interest in testing the resilience of the pathway to a combination of system pressures, which would simultaneously reduce available flex capacity (given outreach of other specialties) and increase discharge delays to downstream community services (due to reduced availability). Following a sensitivity analysis (Supplementary Material E), modelled results provided some reassurance to decision makers regarding the stability of the pathway in all but the most extreme scenarios.

### Practical considerations

When modelling the stroke pathway – in perhaps the simplest sense – one is either calculating the capacity for a desired performance or calculating the performance for a given capacity. In calculating the number of beds to ensure only 1 in 50 stroke presentations have to wait, Monks et al. [13] provide an example of the former. Our work serves as an example of the latter, with the maximum flex capacity determined in advance of the modelling by the physical size and layout of the hospital wards. In addition to deriving the expected utilisation of this given flex capacity, modelling was used to determine its performance. This revealed that 1 in 100 HASU arrivals would have to wait (Table 1), which was considered tolerable by the stroke centralisation Programme Board. For other investigators seeking to model the stroke pathway, careful consideration should be given to both sought performance as well as any constraints on resources. 

On resources, consideration should also be given to the workforce and diagnostic capability required to support the modelled beds. In this regard, attention should focus on the distribution of bed occupancy (Fig. 3) in addition to bed capacity, given that not all beds will be occupied at all times. The precise amounts (of human and diagnostic resource) will depend on the consequences of having too little at peak times versus the financial costs of having too much when the units are less busy. A full economic analysis was beyond the scope of this current study, although would serve as potentially valuable future work. This could extend to considering the *economies of scale* in pooling units, whereby fewer resources are required to achieve a similar performance. An example of this is available from the current analysis (Table 1), in which greater capacity is required to achieve an equivalent proportion of HASU arrivals having to wait (1.1%) with two ASUs (Option 2) than with one ASU (Option 1). Such assessments should be made alongside consideration of factors such as travel time – both for the patient, in ensuring the crucial window for immediate clinical intervention can be met, as well as for visiting family and friends.

This work has the potential to inform a change in culture for stroke capacity planning both in the UK and further afield where similar models of care and bed modelling practices exist (e.g. Italy [19] and the Netherlands [18, 22, 23]). Through incorporating flex capacity within stochastic models covering different settings of care and provider locations, steps can be taken towards improved whole-system capacity management; with individual care settings considered not in isolation but in a way in which the wider effects of any specific actions are more realistically accounted for. While this study has focussed on application to stroke care, it is possible that other acute clinical pathways may also be appropriate for consideration through a flex capacity approach to capacity management and modelling. This may represent further work for future investigators. Further work being planned within the BNSSG system will, as part of a wider evaluation, assess the extent to which actual data aligns with modelled performance when the centralised pathway goes live.

## Conclusions

At certain times, acute stroke services will need to reach beyond their allocated capacities in order to maintain pathway fluidity. This is especially likely if averages-based approaches have been used in setting allocated capacity. Given the continued and widespread use of such approaches, it is important to have some assessment of how much flexibility is required and how often. If due consideration is not given, hospital bed pools may become overwhelmed and patient outcomes may suffer. Despite these potential negative consequences, relevant evidence and appropriate methodological solutions remain sparse. Through this study, we provide a detailed account of the problem and present a possible solution through robust consideration of flex capacity via computer modelling.

## Supplementary Information

**Additional file 1. Supplementary Material A **Strengthening the Reporting of Empirical Simulation Studies (STRESS) Discrete-event simulation guidelines STRESS-DES.** Supplementary Material B Table SM.B.1.** Specification of parameter assumptions used for modelling the two proposed centralised stroke pathway options. Note that P0, P1, P1+ and P3 refer to discharge destinations within community ‘step-down’ services operating the Discharge-to-Assess (D2A) model of care used in England’s NHS (P0 is usual residence without support; P1 is usual residence with home visits; P1+ is P1 with additional social care support; P3 is a bedded placement within a care home).** Supplementary Material C Figure SM.C.1.** Modelled bed occupancy for the proposed future centralised stroke service, under the (preferred) Option 1. The dashed vertical lines represent the demarcation between allocated and flex capacity utilisation. It is assumed here that there is no flex capacity available for the Rehab units.** Supplementary Material D Table SM.D.1. **Modelled performance results for the proposed future centralised stroke service, involving either one (Option 1; preferred option) or two Acute Stroke Units (Option 2). It is assumed here that there is no flex capacity available.** Supplementary Material E** **Table.SM.E.1** contains the ‘baseline’ results (i.e. with no adjustment to the parameters), which are equivalent to those presented in the main paper. The others contain the results associated with various perturbations to the model parameters, conducted as part of the sensitivity analysis performed.** Table.SM.E.1. **Baseline results.** Table.SM.E.2. **Variations to the fixed delays from Rehab units to the D2A P3 service (baseline = 1.5 day delay).** Table.SM.E.3. **Variations to the HASU length of stay (LOS) assumed for mimic patients (baseline = 1 day LOS).** Table.SM.E.4. **Variations to the fixed delays from Rehab units to the D2A P0/1 service (baseline = 0.25 day delay).** Table.SM.E.5. **System pressure scenario, including 3-day fixed delay from Rehab units to the D2A P3 service (baseline = 1.5 days), 2-day HASU length of stay (LOS) assumed for mimic patients (baseline = 1 day), 1 day fixed delay from Rehab units to the D2A P0/1 service (baseline = 0.25 day delay), and 2 ‘flex beds’ available at Rehab (baseline = 5 ‘flex beds’).

## Data Availability

The data that support the findings of this study are available from NHS Digital but restrictions apply to the availability of these data, which were used under license for the current study, and so are not publicly available. Data are however available from the authors upon reasonable request and with permission of NHS Digital.

## Abbreviations

ASU: Acute stroke unit
BNSSG: Bristol, North Somerset, and South Gloucestershire
ICB: Integrated Care Board
HASU: Hyper acute stroke unit
PAS: Patient Administration System
