# Supplementary material for: Optimising acute stroke pathways through flexible use of bed capacity: a computer modelling study
Source: BMC Health Serv Res. 2022 Aug 20;22:1068. doi: 10.1186/s12913-022-08433-0 (PMC9392305; doi:10.1186/s12913-022-08433-0)
Supplement: Supplementary file 1 — Additional file 1. Supplementary Material A Strengthening the Reporting of Empirical Simulation Studies (STRESS) Discrete-event simulation guidelines STRESS-DES. Supplementary Material B Table SM.B.1. Specification of parameter assumptions used for modelling the two proposed centralised stroke pathway options. Note that P0, P1, P1+ and P3 refer to discharge destinations within community ‘step-down’ services operating the Discharge-to-Assess (D2A) model of care used in England’s NHS (P0 is usual residence without support; P1 is usual residence with home visits; P1+ is P1 with additional social care support; P3 is a bedded placement within a care home). Supplementary Material C Figure SM.C.1. Modelled bed occupancy for the proposed future centralised stroke service, under the (preferred) Option 1. The dashed vertical lines represent the demarcation between allocated and flex capacity utilisation. It is assumed here that there is no flex capacity available for the Rehab units. Supplementary Material D Table SM.D.1. Modelled performance results for the proposed future centralised stroke service, involving either one (Option 1; preferred option) or two Acute Stroke Units (Option 2). It is assumed here that there is no flex capacity available. Supplementary Material E Table.SM.E.1 contains the ‘baseline’ results (i.e. with no adjustment to the parameters), which are equivalent to those presented in the main paper. The others contain the results associated with various perturbations to the model parameters, conducted as part of the sensitivity analysis performed. Table.SM.E.1. Baseline results. Table.SM.E.2. Variations to the fixed delays from Rehab units to the D2A P3 service (baseline = 1.5 day delay). Table.SM.E.3. Variations to the HASU length of stay (LOS) assumed for mimic patients (baseline = 1 day LOS). Table.SM.E.4. Variations to the fixed delays from Rehab units to the D2A P0/1 service (baseline = 0.25 day delay). Table.SM.E.5. System pressure scenario, including [file 12913_2022_8433_MOESM1_ESM.docx]

**Supplementary Material A**

Strengthening the Reporting of Empirical Simulation Studies (STRESS)

Discrete-event simulation guidelines STRESS-DES

| **Section/Subsection** | **Item** | **Recommendation** | | **Submitted paper** |
| --- | --- | --- | --- | --- |
| **Objectives** |  |  | |  |
| Purpose of the model | 1.1 | Explain the background and objectives for the model. | | Optimising capacity along clinical pathways is essential to avoid severe hospital pressure and help ensure best patient outcomes and financial sustainability. Yet, typical approaches, using only average arrival rate and average lengths of stay, are known to underestimate the number of beds required. This study aimed to investigate the extent to which averages-based estimates can be complemented by a robust assessment of additional ‘flex capacity’ requirements, to be used at times of peak demand. |
| Model Outputs | 1.2 | Define all quantitative performance measures that are reported, using equations where necessary. Specify how and when they are calculated during the model run along with how any measures of error such as confidence intervals are calculated. | | Our aim was to study flex capacity utilisation over the simulation period. Specifically, we calculated the following metrics:   - Mean total occupancy, beds - Time within allocated capacity, % - Mean flex capacity required, beds - Time at full allocated and flex capacity, % - Time spent at different levels of occupancy for each unit under consideration, % |
| Experimentation Aims | 1.3 | If the model has been used for experimentation, state the objectives that it was used to investigate.     1. Scenario based analysis – Provide a name and description for each scenario, providing a rationale for the choice of scenarios and ensure that item 2.3 (below) is completed. 2. Design of experiments – Provide details of the overall design of the experiments with reference to performance measures and their parameters (provide further details in *data* below). 3. Simulation Optimisation – (if appropriate) Provide full details of what is to be optimised, the parameters that were included and the algorithm(s) that was be used. Where possible provide a citation of the algorithm(s). | | Scenario based analysis. Full details of scenarios included in the sub-section Study Settings of the Methods section. In sum, we studied three stroke pathways: the current decentralised pathway (A), and the proposed centralised stroke pathway options involving one hyper acute stroke unit, either one (B) or two (C) acute stroke units, and two rehabilitation wards. |
| **Logic** |  |  | |  |
| Base model overview diagram | 2.1 | Describe the base model using appropriate diagrams and description. This could include one or more process flow, activity cycle or equivalent diagrams sufficient to describe the model to readers. Avoid complicated diagrams in the main text. The goal is to describe the breadth and depth of the model with respect to the system being studied. | | 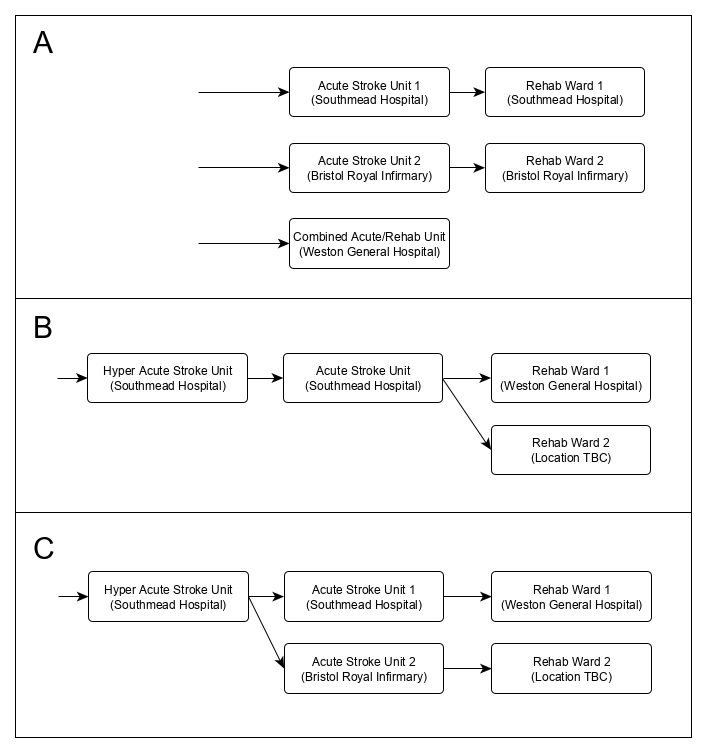 |
| Base model logic | 2.2 | Give details of the base model logic. Give additional model logic details sufficient to communicate to the reader how the model works. | | In the base model, acute stroke care is decentralised and is commissioned from the three hospitals operating within the BNSSG geography, with ongoing rehabilitation commissioned from the single community services provider (Figure 2A). |
| Scenario logic | 2.3 | Give details of the logical difference between the base case model and scenarios (if any). This could be incorporated as text or where differences are substantial could be incorporated in the same manner as 2.2. | | The difference between the base case model (A) and the scenarios (B and C) is in the structure of the care pathway, see section Methods for more details. |
| Algorithms | 2.4 | Provide further detail on any algorithms in the model that (for example) mimic complex or manual processes in the real world (i.e. scheduling of arrivals/appointments/operations/maintenance, operation of a conveyor system, machine breakdowns, etc.). Sufficient detail should be included (or referred to in other published work) for the algorithms to be reproducible. Pseudo-code may be used to describe an algorithm. | | Implementation of this model is through the iterative three-phased method of discrete event simulation (Pidd, 1998). Separate ‘discrete’ events are generated according to a schedule in which the next unconditional event is executed alongside any associated conditional events. The first type of unconditional event is a patient arrival, i.e., a stroke presentation at the HASU. If this occurs at a time when there is sufficient HASU capacity, then the generated conditional event is HASU admission. Otherwise, the patient must wait. The second type of unconditional event is a patient becoming ready for discharge from one of the pathway units. If there is available capacity at the discharge destination then they are discharged, and any upstream waiting patient is admitted in their stead. Otherwise, they remain at their current location until downstream capacity becomes available. The schedule is updated at each iteration and events continue until the end of the simulation period is reached. |
| Components | 2.5 | 2.5.1 Entities | Give details of all entities within the simulation including a description of their role in the model and a description of all their attributes. | Individual patients, each patient has an arrival time and a planned duration of care in the different units (i.e., HASU, ASU, Rehab 1, Rehab 2) time as sampled from the appropriate length of stay distribution.  Full details are provided in section Computer Simulation of the paper and Supplementary Material A. |
|  |  | 2.5.2 Activities | Describe the activities that entities engage in within the model. Provide details of entity routing into and out of the activity. | Arrival, start of service in the different units of care, transfer between units and discharge from the service. |
|  |  | 2.5.3 Resources | List all the resources included within the model and which activities make use of them. | There resources used by simulated patients in the model are beds in one of the care units. |
|  |  | 2.5.4 Queues | Give details of the assumed queuing discipline used in the model (e.g. First in First Out, Last in First Out, prioritisation, etc.). Where one or more queues have a different discipline from the rest, provide a list of queues, indicating the queuing discipline used for each. If reneging, balking or jockeying occur, etc., provide details of the rules. Detail any delays or capacity constraints on the queues. | There are assumed queues linked to each of the care units in the different models (base, suggested options). The discipline of all queues is FIFO. There is no reneging, balking or jockeying. A patient waiting for downstream resource availability remain at their current location until suitable resource (i.e., bed) becomes available. |
|  |  | 2.5.5 Entry/Exit Points | Give details of the model boundaries i.e. all arrival and exit points of entities. Detail the arrival mechanism (e.g. ‘thinning’ to mimic a non-homogenous Poisson process or balking) | Entry: patient arrival to HASU (Poisson distributed).  Exit point: discharged from any of the care units included in the model (e.g. ‘mimics’ for a number of reasons including: when a suspected stroke was not confirmed (mimics); repatriation to other acute hospital; death. |
| **Data** |  |  | |  |
| Data sources | 3.1 | List and detail all data sources. Sources may include:   - Interviews with stakeholders, - Samples of routinely collected data, - Prospectively collected samples for the purpose of the simulation study, - Public domain data published in either academic or organisational literature. Provide, where possible, the link and DOI to the data or reference to published literature.   All data source descriptions should include details of the sample size, sample date ranges and use within the study. | | Given that, at the time of the study, many acute stroke services were non-centralised, there was limited empirical data available to support model calibration, especially regarding the upstream part of the pathway including the HASU. Where possible, the relevant information was used with downstream parameters estimated using local data obtained from hospital Patient Administration Systems (PAS) and from a 2017 service evaluation. This service evaluation was performed specifically to support the capacity modelling. Detailed information was captured that is not collected within the PAS, e.g. date medically fit for discharge – useful in calculating length of stay until the point of discharge readiness. All model parameters for both Options 1 and 2 are contained in full in Table SM.A.1 in the Supplementary document, including the specific source of information used for estimation. |
| Pre-processing | 3.2 | Provide details of any data manipulation that has taken place before its use in the simulation, e.g. interpolation to account for missing data or the removal of outliers. | | N/A |
| Input parameters | 3.3 | List all input variables in the model. Provide a description of their use and include parameter values. For stochastic inputs provide details of any continuous, discrete or empirical distributions used along with all associated parameters. Give details of all time dependent parameters and correlation.  Clearly state:   - Base case data - Data use in experimentation, where different from the base case. - Where optimisation or design of experiments has been used, state the range of values that parameters can take.   Where theoretical distributions are used, state how these were selected and prioritised above other candidate distributions. | | See Table SM.A.1 in the Supplementary document. |
| Assumptions | 3.4 | Where data or knowledge of the real system is unavailable what assumptions are included in the model? This might include parameter values, distributions or routing logic within the model. | | See subsection Strengths and limitation in the Discussion. |
| **Experimentation** |  |  | |  |
| Initialisation | 4.1 | Report if the system modelled is terminating or non-terminating. State if a warm-up period has been used, its length and the analysis method used to select it. For terminating systems state the stopping condition.  State what if any initial model conditions have been included, e.g., pre-loaded queues and activities. Report whether initialisation of these variables is deterministic or stochastic. | | Non-terminating system with warm-up period of 100 days. No initial conditions have been used. |
| Run length | 4.2 | Detail the run length of the simulation model and time units. | | Time unit is day. Each run was one year long. |
| Estimation approach | 4.3 | State the method used to account for the stochasticity: For example, two common methods are multiple replications or batch means. Where multiple replications have been used, state the number of replications and for batch means, indicate the batch length and whether the batch means procedure is standard, spaced or overlapping. For both procedures provide a justification for the methods used and the number of replications/size of batches. | | 1500 independent replications were used for each simulated scenario. |
| **Implementation** |  |  | |  |
| Software or programming language | 5.1 | State the operating system and version and build number.  State the name, version and build number of commercial or open source DES software that the model is implemented in.  State the name and version of general-purpose programming languages used (e.g. Python 3.5).  Where frameworks and libraries have been used provide all details including version numbers. | | The model was coded from scratch in R and has been released as an open-source tool (hosted on <https://github.com/nhs-bnssg-analytics/PathSimR> and promoted via social media). |
| Random sampling | 5.2 | State the algorithm used to generate random samples in the software/programming language used e.g. Mersenne Twister.  If common random numbers are used, state how seeds (or random number streams) are distributed among sampling processes. | | Uses the inbuilt random number generator in R. Each replication uses a different seed call to this function. This provides the necessary stochastic variation within each replication, yet also allows reproducible model scenarios to be created and assessed (useful when evaluating specific changes in the model parameters). |
| Model execution | 5.3 | State the event processing mechanism used e.g. three phase, event, activity, process interaction.  *Note that in some commercial software the event processing mechanism may not be published. In these cases authors should adhere to item 5.1 software recommendations.*  State all priority rules included if entities/activities compete for resources.  If the model is parallel, distributed and/or use grid or cloud computing, etc., state and preferably reference the technology used. For parallel and distributed simulations the time management algorithms used. If the HLA is used then state the version of the standard, which run-time infrastructure (and version), and any supporting documents (FOMs, etc.) | | The tool implements the established ‘three phase’ method to stochastic simulation [28], in which separate ‘discrete’ events are generated according to a schedule in which the next unconditional event is executed alongside any associated conditional events. The first type of unconditional event is a patient arrival, i.e. a stroke presentation at the HASU. If this occurs at a time when there is sufficient HASU capacity, then the generated conditional event is HASU admission. Otherwise, the patient must wait. The second type of unconditional event is a patient becoming ready for discharge from one of the pathway units. If there is available capacity at the discharge destination then they are discharged, and any upstream waiting patient is admitted in their stead. Otherwise, they remain at their current location until downstream capacity becomes available. The schedule is updated at each iteration and events continue until the end of the simulation period is reached. |
| System Specification | 5.4 | State the model run time and specification of hardware used. This is particularly important for large scale models that require substantial computing power. For parallel, distributed and/or use grid or cloud computing, etc. state the details of all systems used in the implementation (processors, network, etc.) | | Processing time is insubstantial, typically taking less than five minutes for each scenario evaluated on a desktop computer. |
| **Code Access** |  |  | |  |
| Computer Model Sharing Statement | 6.1 | Describe how someone could obtain the model described in the paper, the simulation software and any other associated software (or hardware) needed to reproduce the results. Provide, where possible, the link and DOIs to these. | | The tool is open source and available for free: <https://github.com/nhs-bnssg-analytics/PathSimR> |

**Supplementary Material B**

**Table SM.B.1.** Specification of parameter assumptions used for modelling the two proposed centralised stroke pathway options. Note that P0, P1, P1+ and P3 refer to discharge destinations within community ‘step-down’ services operating the Discharge-to-Assess (D2A) model of care used in England’s NHS (P0 is usual residence without support; P1 is usual residence with home visits; P1+ is P1 with additional social care support; P3 is a bedded placement within a care home).

| **Unit** | **Measure** | **Value** | **Source** |
| --- | --- | --- | --- |
| HASU | Arrival rate | 6.4 per day* | Combination of:  - Local ambulance trust (SWAST) data from 2019/20 for patients with suspected stroke conveyed to BNSSG hospitals  - Estimated proportion of stroke mimics arriving on the HASU  - SSNAP Team-Centred 72 hours data for 2019/20  - Number of mechanical thrombectomy transfers to Southmead from outside BNSSG for 2019/20  - 5% growth |
| HASU | Allocated capacity | 22 beds | Based on averages-based calculation (see Background; Flex Capacity) |
| HASU | Flex capacity | 10 beds | Based on maximum physical ward capacity of 32 beds (i.e. 32 minus allocated capacity) |
| HASU | Mean length of stay | 3 days** | Based on combined LOS for mimics (1.0 days, estimated from local audit, research evidence and adjusted for improved access to same-day emergency care and imaging) and stroke (3.5 days based on London HASU LOS from SSNAP 2019/20) |
| HASU | Transfer – P0/P1 | 22% | SSNAP London HASU data: % of those discharged alive discharged home with or without ESD/CRT |
| HASU | Exit pathway – mimics | 20% | Based on mimic percentage of 25% within BNSSG (derived from Southmead mimic audit and research evidence: St George’s Hospital, London – Dawson et al, 2016), which becomes 20% when non-BNSSG thrombectomy is included |
| HASU | Exit pathway – repatriation to other acute hospital | 8% | Non-BNSSG thrombectomy repats plus London HASU SSNAP Transfer Tree transfers to acute hospitals off usual pathway |
| HASU | Exit pathway – death | 8% | Based on combined mortality for stroke in general (10%, based on London HASUs), thrombectomy (15%) and mimics (0%) |
| HASU | Fixed delay – repatriation to other acute hospital | 1.5 days | Based on processes required for repatriation: referral, acceptance, transfer (no delays) |
| HASU | Fixed delay – P0/P1 | 0.25 days | Processes required for discharge, no further delay |
| ASU, ASU 1 / 2 | Mean length of stay | 6 days*** | Southmead Hospital “Flow” data: admission to medically fit for discharge, cross-referenced with 2017 BNSSG ‘audit’ total acute LOS (HASU+ASU) |
| ASU, ASU 1 / 2 | Transfer – P0/P1 | 36% | Based on total proportion discharged home from acute (HASU+ASU) determined from 2017 BNSSG ‘audit’ |
| ASU, ASU 1 / 2 | Transfer – Rehab 1 | 33.5% | Total Rehab 1 + Rehab 2 determined by 2017 ‘audit’; split according to postcode |
| ASU, ASU 1 / 2 | Transfer – Rehab 2 | 13.5% | Total Rehab 1 + Rehab 2 determined by 2017 “audit”; split according to postcode |
| ASU, ASU 1 / 2 | Exit pathway – repatriation to other acute hospital | 10% | Check of individual postcodes (non-BNSSG postcodes) from SSNAP |
| ASU, ASU 1 / 2 | Exit pathway – death | 7% | Set to make combined acute mortality (HASU+ASU) for BNSSG patients match BNSSG mortality from SSNAP |
| ASU, ASU 1 / 2 | Fixed delay – repatriation to other acute hospital | 1.5 days | Processes required for repat, no further delay |
| ASU, ASU 1 / 2 | Fixed delay – Rehab 1 | 1.5 days | Processes required for discharge, no further delay |
| ASU, ASU 1 / 2 | Fixed delay – Rehab 2 | 1.5 days | Processes required for discharge, no further delay |
| Rehab 1 | Allocated capacity | 30 beds | Based on averages-based calculation, split between two rehab units determined according to postcode |
| Rehab 2 | Allocated capacity | 12 beds | Based on averages-based calculation, split between two rehab units determined according to postcode |
| Rehab 1 | Flex capacity | 5 beds | Chosen based on distribution of bed occupancy for stochastic model with no upper capacity limit |
| Rehab 2 | Flex capacity | 5 beds | Chosen based on distribution of bed occupancy for stochastic model with no upper capacity limit |
| Rehab 1 / 2 | Mean length of stay | 26 days**** | 2017 BNSSG ‘audit’, adjusted for planned therapy service improvements, with total pathway LOS checked against SSNAP total stroke unit LOS for other centres |
| Rehab 1 / 2 | Transfer – P0/P1 | 19% | 2017 BNSSG ‘audit’, home from subacute phase of care |
| Rehab 1 / 2 | Transfer – P1+ | 50% | 2017 BNSSG ‘audit’, ongoing rehab from subacute phase of care |
| Rehab 1 / 2 | Transfer – P3 | 31% | 2017 BNSSG ‘audit’ |
| Rehab 1 / 2 | Fixed delay – P0/P1 | 0.25 days | Processes required for discharge, no further delay |
| Rehab 1 / 2 | Fixed delay – P1+ | 1.5 days | Processes required for discharge (including social care), no further delay |
| Rehab 1 / 2 | Fixed delay – P3 | 1.5 days | Processes required for discharge to P3, no further delay |
| **Relevant only to Option 1** | | | |
| HASU | Transfer to ASU | 42% | Based on SSNAP Transfer Tree for London HASU with closest pathway to proposed future BNSSG model |
| ASU | Allocated capacity | 22 beds | Based on averages-based calculation |
| ASU | Flex capacity | 10 beds | Based on maximum physical ward capacity of 32 beds (i.e. 32 minus allocated capacity) |
| **Relevant only to Option 2** | | | |
| HASU | Transfer to ASU 1 | 25% | 42% above, split according to postcode |
| HASU | Transfer to ASU 2 | 17% | 42% above, split according to postcode |
| ASU 1 | Allocated capacity | 15 beds | Based on averages-based calculation, split by postcode |
| ASU 2 | Allocated capacity | 9 beds | Based on averages-based calculation, split by postcode |
| ASU 1 | Flex capacity | 17 beds | Based on 32 bedded ward |
| ASU 2 | Flex capacity | 11 beds | Based on current acute stroke bed base at ASU 2 hospital (Bristol Royal Infirmary): 20 beds (9+11) |

* Poisson distributed (λ = 6.4).

** Exponentially distributed ($\mu$ = 0.3333).

*** Exponentially distributed ($\mu$ = 0.1667).

**** Exponentially distributed ($\mu$ = 0. 0384).

**Supplementary Material C**


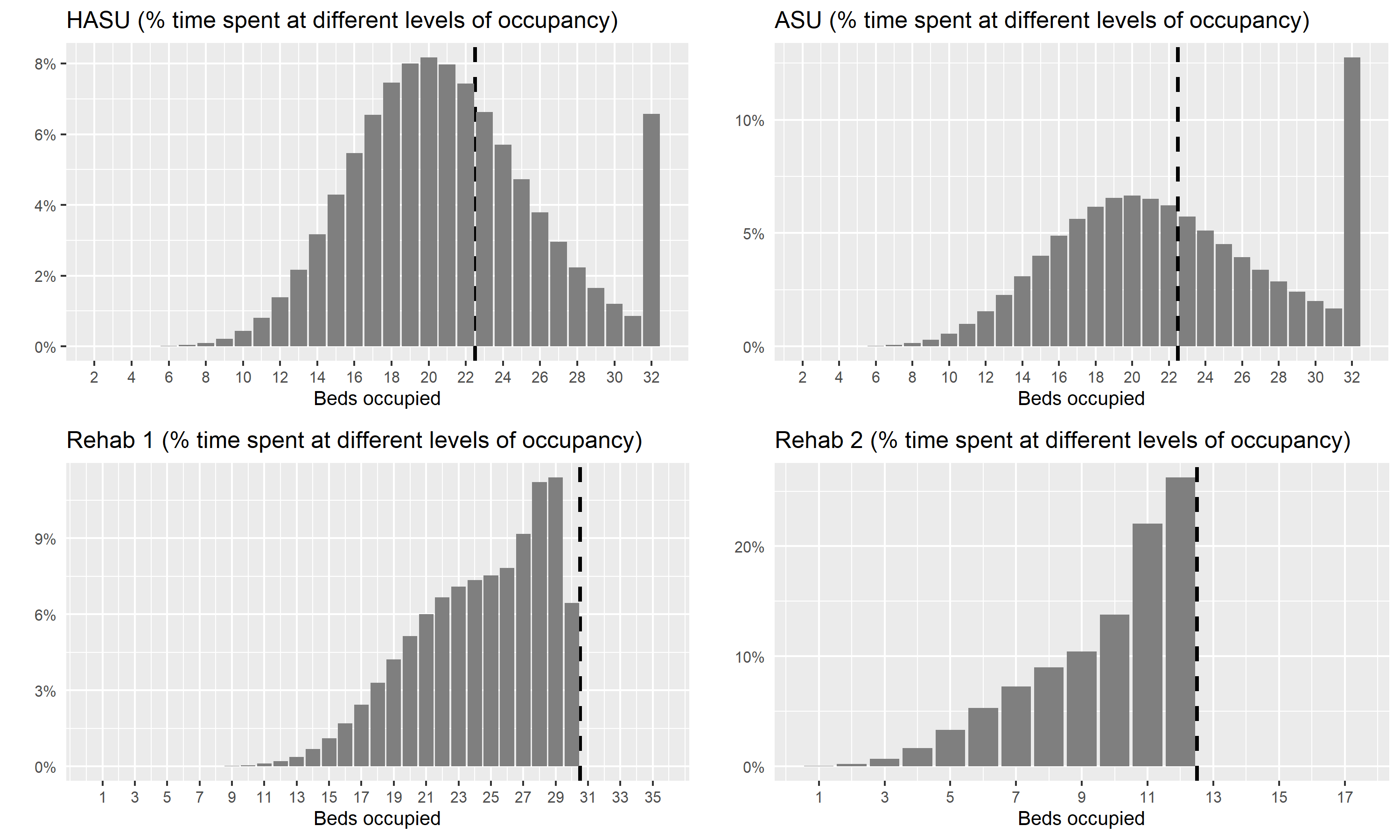


**Figure SM.C.1.** Modelled bed occupancy for the proposed future centralised stroke service, under the (preferred) Option 1. The dashed vertical lines represent the demarcation between allocated and flex capacity utilisation. It is assumed here that there is no flex capacity available for the Rehab units.

**Supplementary Material D**

**Table SM.D.1.** Modelled performance results for the proposed future centralised stroke service, involving either one (Option 1; preferred option) or two Acute Stroke Units (Option 2). It is assumed here that there is no flex capacity available.

| **Option** | **Unit** | **Allocated capacity, beds** | **Mean total occupancy, beds** | **Time at full allocated, %** |
| --- | --- | --- | --- | --- |
| 1 | HASU | 22 | 21.5 | 89.2 |
|  | ASU | 22 | 19.8 | 50.8 |
|  | Rehab 1 | 30 | 23.7 | 4.9 |
|  | Rehab 2 | 12 | 9.5 | 22.6 |
| 2 | HASU | 22 | 21.8 | 94.4 |
|  | ASU 1 | 15 | 11.5 | 26.5 |
|  | ASU 2 | 9 | 7.8 | 58.1 |
|  | Rehab 1 | 30 | 23.3 | 4.2 |
|  | Rehab 2 | 12 | 9.3 | 21 |

**Supplementary Material E**

Table.SM.E.1 contains the ‘baseline’ results (i.e. with no adjustment to the parameters), which are equivalent to those presented in the main paper. The others contain the results associated with various perturbations to the model parameters, conducted as part of the sensitivity analysis performed.

| **Scenario** | **Unit** | **Beds** | **Mean total occupancy, beds** | **Time within allocated capacity, %** | **Mean flex capacity required, beds** | **Time at full allocated and flex capacity, %** |
| --- | --- | --- | --- | --- | --- | --- |
| Option 1 | HASU | 22 (32) | 20.3 | 69.6 | 3.6 | 1.1 |
| Option 1 | ASU | 22 (32) | 18.9 | 79.9 | 3.3 | 0.5 |
| Option 1 | Rehab 1 | 30 (35) | 24.4 | 88.2 | 2.5 | 0.9 |
| Option 1 | Rehab 2 | 12 (17) | 9.9 | 79.1 | 2.4 | 2.1 |
| Option 2 | HASU | 22 (32) | 20.3 | 69.5 | 3.4 | 1.1 |
| Option 2 | ASU 1 | 15 (32) | 11.2 | 89.2 | 2.5 | 0 |
| Option 2 | ASU 2 | 9 (20) | 7.6 | 75.9 | 2.4 | 0 |
| Option 2 | Rehab 1 | 30 (35) | 24.5 | 87.9 | 2.5 | 0.9 |
| Option 2 | Rehab 2 | 12 (17) | 9.9 | 79.8 | 2.4 | 1.8 |

**Table.SM.E.1.** Baseline results.

| **Scenario** | **Unit** | **Beds** | **Mean total occupancy, beds** | **Time within allocated capacity, %** | **Mean flex capacity required, beds** | **Time at full allocated and flex capacity, %** |
| --- | --- | --- | --- | --- | --- | --- |
| Option 1 (3 day delay) | HASU | 22 (32) | 20.3 | 69.5 | 3.6 | 1.2 |
| Option 1 (3 day delay) | ASU | 22 (32) | 18.9 | 79.9 | 3.4 | 0.7 |
| Option 1 (3 day delay) | Rehab 1 | 30 (35) | 24.8 | 86.6 | 2.1 | 1.1 |
| Option 1 (3 day delay) | Rehab 2 | 12 (17) | 10 | 78.8 | 2.4 | 2 |
| Option 2 (3 day delay) | HASU | 22 (32) | 20.3 | 69.3 | 3.6 | 1.1 |
| Option 2 (3 day delay) | ASU 1 | 15 (32) | 11.3 | 88.9 | 2.5 | 0 |
| Option 2 (3 day delay) | ASU 2 | 9 (20) | 7.7 | 75.2 | 2.4 | 0.1 |
| Option 2 (3 day delay) | Rehab 1 | 30 (35) | 24.9 | 85.8 | 2.6 | 1.2 |
| Option 2 (3 day delay) | Rehab 2 | 12 (17) | 10 | 78.5 | 2.4 | 2 |
| Option 1 (7 day delay) | HASU | 22 (32) | 20.4 | 69.1 | 3.6 | 1.3 |
| Option 1 (7 day delay) | ASU | 22 (32) | 19.2 | 77.7 | 3.6 | 1.1 |
| Option 1 (7 day delay) | Rehab 1 | 30 (35) | 26.1 | 79.2 | 2.5 | 2.1 |
| Option 1 (7 day delay) | Rehab 2 | 12 (17) | 10.5 | 73.4 | 2.5 | 2.7 |
| Option 2 (7 day delay) | HASU | 22 (32) | 20.4 | 68.8 | 3.6 | 1.2 |
| Option 2 (7 day delay) | ASU 1 | 15 (32) | 11.4 | 88.2 | 2.6 | 0 |
| Option 2 (7 day delay) | ASU 2 | 9 (20) | 7.7 | 74.6 | 2.5 | 0.1 |
| Option 2 (7 day delay) | Rehab 1 | 30 (35) | 25.9 | 80.4 | 2.7 | 1.9 |
| Option 2 (7 day delay) | Rehab 2 | 12 (17) | 10.5 | 72.7 | 2.5 | 3.1 |

**Table.SM.E.2.** Variations to the fixed delays from Rehab units to the D2A P3 service (baseline = 1.5 day delay).

| **Scenario** | **Unit** | **Beds** | **Mean total occupancy, beds** | **Time within allocated capacity, %** | **Mean flex capacity required, beds** | **Time at full allocated and flex capacity, %** |
| --- | --- | --- | --- | --- | --- | --- |
| Option 1 (2 day LOS) | HASU | 22 (32) | 21.6 | 58.8 | 3.3 | 2.3 |
| Option 1 (2 day LOS) | ASU | 22 (32) | 18.9 | 80.2 | 3.3 | 0.5 |
| Option 1 (2 day LOS) | Rehab 1 | 30 (35) | 24.4 | 88.7 | 1.8 | 0.8 |
| Option 1 (2 day LOS) | Rehab 2 | 12 (17) | 9.9 | 79.7 | 1.6 | 1.8 |
| Option 2 (2 day LOS) | HASU | 22 (32) | 21.6 | 58.6 | 4 | 2.6 |
| Option 2 (2 day LOS) | ASU 1 | 15 (32) | 11.2 | 89.3 | 2.5 | 0 |
| Option 2 (2 day LOS) | ASU 2 | 9 (20) | 7.7 | 75.8 | 2.4 | 0 |
| Option 2 (2 day LOS) | Rehab 1 | 30 (35) | 24.4 | 88.3 | 2.5 | 0.9 |
| Option 2 (2 day LOS) | Rehab 2 | 12 (17) | 9.8 | 80.1 | 2.4 | 1.7 |
| Option 1 (2 day LOS) | HASU | 22 (32) | 24.2 | 37.2 | 5.1 | 8.9 |
| Option 1 (4 day LOS) | ASU | 22 (32) | 19 | 79.2 | 3.4 | 0.7 |
| Option 1 (4 day LOS) | Rehab 1 | 30 (35) | 24.5 | 87.8 | 2.5 | 0.9 |
| Option 1 (4 day LOS) | Rehab 2 | 12 (17) | 9.9 | 80 | 2.4 | 1.7 |
| Option 1 (4 day LOS) | HASU | 22 (32) | 24.2 | 37.2 | 5.1 | 8.8 |
| Option 2 (4 day LOS) | ASU 1 | 15 (32) | 11.2 | 89.2 | 2.4 | 0 |
| Option 2 (4 day LOS) | ASU 2 | 9 (20) | 7.6 | 76.3 | 2.4 | 0 |
| Option 2 (4 day LOS) | Rehab 1 | 30 (35) | 24.6 | 87.8 | 2.5 | 1 |
| Option 2 (4 day LOS) | Rehab 2 | 12 (17) | 9.8 | 80.4 | 2.4 | 1.8 |

**Table.SM.E.3.** Variations to the HASU length of stay (LOS) assumed for mimic patients (baseline = 1 day LOS).

| **Scenario** | **Unit** | **Beds** | **Mean total occupancy, beds** | **Time within allocated capacity, %** | **Mean flex capacity required, beds** | **Time at full allocated and flex capacity, %** |
| --- | --- | --- | --- | --- | --- | --- |
| Option 1 (0.5 day delay) | HASU | 22 (32) | 20.7 | 66.4 | 3.7 | 1.4 |
| Option 1 (0.5 day delay) | ASU | 22 (32) | 19.1 | 78.4 | 3.3 | 0.7 |
| Option 1 (0.5 day delay) | Rehab 1 | 30 (35) | 24.4 | 88.5 | 2.5 | 0.8 |
| Option 1 (0.5 day delay) | Rehab 2 | 12 (17) | 10 | 78.9 | 2.4 | 1.9 |
| Option 2 (0.5 day delay) | HASU | 22 (32) | 20.7 | 66.6 | 3.7 | 1.4 |
| Option 2 (0.5 day delay) | ASU 1 | 15 (32) | 11.4 | 88.2 | 2.5 | 0 |
| Option 2 (0.5 day delay) | ASU 2 | 9 (20) | 7.8 | 75 | 2.5 | 0 |
| Option 2 (0.5 day delay) | Rehab 1 | 30 (35) | 24.7 | 87.6 | 2.5 | 1 |
| Option 2 (0.5 day delay) | Rehab 2 | 12 (17) | 9.9 | 80 | 2.4 | 1.8 |
| Option 1 (1 day delay) | HASU | 22 (32) | 21.4 | 60.9 | 3.9 | 2.2 |
| Option 1 (1 day delay) | ASU | 22 (32) | 19.5 | 75.4 | 3.5 | 0.9 |
| Option 1 (1 day delay) | Rehab 1 | 30 (35) | 24.5 | 88 | 2.5 | 0.9 |
| Option 1 (1 day delay) | Rehab 2 | 12 (17) | 9.9 | 79.8 | 2.4 | 1.8 |
| Option 2 (1 day delay) | HASU | 22 (32) | 21.4 | 61 | 3.9 | 2.1 |
| Option 2 (1 day delay) | ASU 1 | 15 (32) | 11.6 | 86.7 | 2.8 | 0 |
| Option 2 (1 day delay) | ASU 2 | 9 (20) | 7.9 | 72.6 | 2.7 | 0 |
| Option 2 (1 day delay) | Rehab 1 | 30 (35) | 24.6 | 86.7 | 2.6 | 1.1 |
| Option 2 (1 day delay) | Rehab 2 | 12 (17) | 9.8 | 79.9 | 2.4 | 1.8 |
| Option 1 (3 day delay) | HASU | 22 (32) | 24.3 | 36.4 | 5.1 | 9.5 |
| Option 1 (3 day delay) | ASU | 22 (32) | 21.6 | 58.8 | 3.1 | 2.9 |
| Option 1 (3 day delay) | Rehab 1 | 30 (35) | 24.9 | 86 | 1.9 | 1.2 |
| Option 1 (3 day delay) | Rehab 2 | 12 (17) | 10.1 | 77.1 | 1.7 | 2.3 |
| Option 2 (3 day delay) | HASU | 22 (32) | 24.2 | 37 | 5.1 | 9 |
| Option 2 (3 day delay) | ASU 1 | 15 (32) | 12.9 | 77.4 | 2.9 | 0 |
| Option 2 (3 day delay) | ASU 2 | 9 (20) | 8.7 | 62.2 | 2.8 | 0.1 |
| Option 2 (3 day delay) | Rehab 1 | 30 (35) | 25 | 85.1 | 2.6 | 1.3 |
| Option 2 (3 day delay) | Rehab 2 | 12 (17) | 10 | 78.6 | 2.4 | 2 |

**Table.SM.E.4.** Variations to the fixed delays from Rehab units to the D2A P0/1 service (baseline = 0.25 day delay).

| **Scenario** | **Unit** | **Beds** | **Mean total occupancy, beds** | **Time within allocated capacity, %** | **Mean flex capacity required, beds** | **Time at full allocated and flex capacity, %** |
| --- | --- | --- | --- | --- | --- | --- |
| Option 1 (sys pressure) | HASU | 22 (32) | 22.8 | 48.4 | 4.6 | 5.5 |
| Option 1 (sys pressure) | ASU | 22 (32) | 20.8 | 65.7 | 4.4 | 4.3 |
| Option 1 (sys pressure) | Rehab 1 | 30 (32) | 25 | 88.2 | 1.3 | 4.1 |
| Option 1 (sys pressure) | Rehab 2 | 12 (14) | 10 | 75.8 | 1.5 | 11.7 |
| Option 2 (sys pressure) | HASU | 22 (32) | 22.7 | 49.8 | 4.4 | 4.4 |
| Option 2 (sys pressure) | ASU 1 | 15 (32) | 12.2 | 81.5 | 3.2 | 0 |
| Option 2 (sys pressure) | ASU 2 | 9 (20) | 8.4 | 67.3 | 2.9 | 0.3 |
| Option 2 (sys pressure) | Rehab 1 | 30 (32) | 24.6 | 90.2 | 2.8 | 3.3 |
| Option 2 (sys pressure) | Rehab 2 | 12 (14) | 9.9 | 77.4 | 3 | 10.8 |

**Table.SM.E.5.** System pressure scenario, including 3-day fixed delay from Rehab units to the D2A P3 service (baseline = 1.5 days), 2-day HASU length of stay (LOS) assumed for mimic patients (baseline = 1 day), 1 day fixed delay from Rehab units to the D2A P0/1 service (baseline = 0.25 day delay), and 2 ‘flex beds’ available at Rehab (baseline = 5 ‘flex beds’).
